# Chronic inflammatory demyelinating polyneuropathy (CIDP): change of serum IgG dimer levels during treatment with intravenous immunoglobulins

**DOI:** 10.1186/s12974-015-0361-1

**Published:** 2015-08-14

**Authors:** Christian Ritter, Ilja Bobylev, Helmar C. Lehmann

**Affiliations:** Department of Neurology, University Hospital Cologne, Cologne, Germany; Center of Molecular Medicine Cologne (CMMC), University of Cologne, Cologne, Germany; Cognitive Neuroscience, Institute of Neuroscience and Medicine (INM-3), Research Centre Jülich, Jülich, Germany

**Keywords:** Immune neuropathy, Surrogate marker, Inflammation, Autoantibody

## Abstract

**Background:**

Intravenous immunoglobulin (IVIg) is an effective treatment in chronic inflammatory demyelinating polyneuropathy (CIDP). In most patients, the optimal IVIg dose and regime is unknown. Polyvalent immunoglobulin (Ig) G form idiotypic/anti-idiotypic antibody pairs in serum and IVIg preparations. We determined IgG dimer levels before and after IVIg treatment in CIDP patients with the aim to explore their utility to serve as a surrogate marker for treatment response.

**Methods:**

IgG was purified from serum of five controls without treatment, as well as from serum of 16 CIDP patients, two patients with Miller Fisher syndrome (MFS), and one patient with myasthenia gravis before and after treatment with IVIg. IgG dimer levels were determined by size exclusion chromatography. IgG dimer formation was correlated with clinical response to IVIg treatment in CIDP. Re-monomerized IgG dimer fractions were analyzed for immunoreactivity against peripheral nerve tissue.

**Results:**

IgG dimer levels were significantly higher in post- compared to pre-IVIg infusion samples. Low post-treatment IgG dimer levels in CIDP patients were associated with clinical worsening during IVIg treatment. Re-monomerized IgG dimer fractions from CIDP patients showed immunoreactivity against peripheral nerve tissue, whereas similarly treated samples from MFS patients showed immunoreactivity against GQ1b.

**Conclusion:**

Assessment of IgG dimer levels could be a novel approach to monitor CIDP patients during IVIg treatment, but further studies in larger cohorts are warranted to explore their utility to serve as a potential therapeutic biomarker for IVIg treatment response in CIDP.

## Background

Chronic inflammatory demyelinating polyneuropathy (CIDP) belongs to the most common immune-mediated chronic disorders of the peripheral nervous system. Abundant experimental evidence suggests that CIDP is caused by an aberrant immune response that involves autoreactive T cells, macrophages, and autoantibodies [1–4]. However, despite extensive research over the last decades, the target antigens of the humoral and cell-mediated immune response are yet poorly defined.

Intravenous immunoglobulin (IVIg) is considered the first-line treatment for CIDP, and several controlled studies demonstrated its short- and long-term efficacy [5–7]. The therapeutic effect of IVIg in CIDP is not fully understood, but it is believed that different modes of action are involved. These mechanisms include blockage or modulation of Fcγ-receptors, regulation of T cell and B cell activation, and alteration of inflammatory cytokines [8–10]. In addition, there is evidence from other autoimmune diseases that IVIg contains anti-idiotypic (anti-Id) antibodies that are able to neutralize pathological idiotypic autoantibodies (Id) by forming Id-anti-Id complexes [11–15].

IVIg preparations contain pooled IgG from different donors and are mostly monomeric IgG. However, a small percentage of polyvalent IgG form Id-anti-Id antibody dimers [16, 17]. Increased levels of IgG dimers have been linked to adverse events during IVIg infusion [18]. In contrast, there are experimental studies that suggest beneficial effects of increased IgG dimer levels in IVIg preparations [19].

Dimeric IgG is also detectable in the serum of healthy individuals, although its amount compared to monomeric IgG is usually much lower than that in IVIg preparations [20, 21]. Notably, it is currently unknown if IVIg treatment influences the circulating IgG dimer content in humans with immune-mediated neurological diseases.

We hypothesized that in CIDP, IVIg treatment may result in the formation of new Id-anti-Id complexes that could be assessed by quantification of Ig dimers in post-treatment serum of CIDP patients. We anticipated that correlations between IgG dimer formation and clinical response in individual patients could serve as a potential surrogate marker for treatment response in CIDP, in which optimal dose and regimen of IVIg is still unknown. Moreover, purified IgG dimers, containing Id-anti-Id complexes, could be employed as a novel approach to identify idiotypic (auto)antibodies and their targeted antigens in CIDP.

## Methods

### Patients

Sixteen patients with CIDP (mean age 64.4 ± 12.2, nine males, seven females) were included in the study. All patients were diagnosed according to diagnostic criteria developed by the Peripheral Nerve Society [22]. Fourteen of 16 patients were on maintenance therapy with IVIg and received a standard dose of 1 g/kg bodyweight (BW). Blood was obtained immediately before and 30 min after IVIg infusion. All patients underwent a detailed clinical workup prior to the IVIg infusion, which also included assessment of the Medical Research Council (MRC) sum score and Inflammatory Neuropathy Cause and Treatment (INCAT) disability score as previously described [23] (Table 1). Patients were considered stable when INCAT score and MRC sum score remained unchanged over 6 months of IVIg treatment, and worsening to IVIg treatment was defined as change of either INCAT or MRC sum score within 6 months.

**Table 1:** Clinical characteristics from CIDP patients with INCAT disability score, MRC sum score, and treatment history

Patient	Sex	Age	Treatment before sampling	IVIg treatment duration before d1 (months)	INCAT score d1	INCAT score 6 months	MRC SS d1	MRC SS 6 months	Dimer content pre-IVIg d1 (%)	Dimer content post-IVIg d1 (%)
1	M	58	CS	0	2	2	58	58	1.3	3.0
2	M	62	CS, IVIg	9	2	2	58	58	3.8	11.3
3	M	67	CS, IVIg	16	1	2	60	60	5.4	5.5
4	M	34	CS, IVIg	10	1	2	52	52	2.4	2.4
5	F	79	CS, IVIg	28	7	6	52	52	4.6	9.3
6	M	61	IVIg	16	2	2	58	58	1.8	4.2
7	M	74	CS, IVIg	27	7	7	46	46	3.7	11.3
8	F	69	IVIg	5	0	0	60	60	2.7	6.0
9	F	73	CS, CP	6	4	7	52	47	2.3	4.1
10	M	48	CS, IVIg	6	3	1	58	58	2.1	3.4
11	F	56	IVIg	2	3	3	58	58	2.1	2.3
12	F	71	IVIg	8	0	0	60	60	3.9	7.4
13	F	79	IVIg	10	2	2	52	52	1,9	7,7
14	M	65	IVIg	10	2	2	58	58	0	4.2
15	F	78	CS, IVIg	12	1	1	58	58	3.7	6.2
16	M	57	-	0	2	2	60	60	0	3.9

*CS* cortisone, *CP* cyclophosphamide, *SS* sum score

In addition, blood was obtained from three patients suffering from other immune-mediated neurological diseases (myasthenia gravis, MG, *n* = 1; Miller Fisher syndrome, MFS, *n* = 2) immediately before and 30 min after IVIg infusion and from five control patients (mean age 51.8 ± 13.9, two females, three males). These included patients with neurological diseases other than CIDP, MG, or MFS (idiopathic cephalgia, peripheral facial nerve paralysis (*n* = 2), subarachnoid hemorrhage, epilepsy).

The study is registered with ClinicalTrials.gov, number NCT01655394, and approved by the local ethics committee (Ethics Committee University of Cologne, #12-182). All patients gave written informed consent prior to inclusion into the study.

### Chromatographic separation of monomeric and dimeric IgG fractions

Blood samples of patients were centrifuged (2000*g*, 15 min), and serum was prior to preparation of IgG fractions diluted 1:4 with low-salt buffer (LS buffer) containing 100 mM HEPES and 10 mM NaCl at pH 7.5. All IgG purification experiments were performed on an automated fast protein liquid chromatography system (AKTA, GE Healthcare Bio-Sciences Corp.) using Unicorn software (GE Healthcare Bio-Sciences Corp.) for analysis.

First, IgG fractions were purified from CIDP serum samples using ion exchange chromatography columns (HighTrap Q HP and HighTrap SP HP, GE Healthcare). Columns were calibrated using LS buffer, and serum samples were injected with a 1 ml sample loop. IgG fractions were dissolved from the HighTrap SP HP column using high-salt (HS) final buffer (100 mM HEPES and 500 mM NaCl at pH 7.5). A gradient was generated over 20 min at a defined flow rate. Collected IgG fractions were re-concentrated to a 1 ml volume using a commercial protein concentration kit (Pierce) according to the manufacturer’s protocol.

The molecular weight (MW) profile of dimeric and monomeric IgG was determined by gel filtration chromatography using an analytical-grade column (Superdex 200, GE Healthcare) and standard gel filtration buffer at a flow rate of 0.5 ml/min.

### SDS-PAGE analysis of monomeric and dimeric IgG fractions

The purity of dimeric and monomeric IgG fractions was checked by sodium dodecyl sulfate-polyacrylamide gel electrophoresis (SDS-PAGE) using nUVview precast Tris-HEPES gels containing 10 % polyacrylamide. Bands were visualized under ultraviolet (UV) light after a 2-min exposure time. As a standard, the low molecular weight (LMW) references from Bio-Rad were used.

### Detection of antibodies against GQ1b and AchR

Commercial ELISA plates (Sigma-Aldrich) were coated with human GQ1b (Sigma-Aldrich). GQ1b-coated microtiter plates were blocked with 1 % BSA solution for 2 h at 4 °C and removed by washing with phosphate-buffered saline (PBS) afterwards. Dimer IgG fraction post-IVIg treatment was re-monomerized by dialyzing against 10 mM acetic acid at pH 4.0 for 24 h. Pre-IVIg treatment monomeric IgG and post-IVIg treatment monomeric and dimeric IgG fractions were added and incubated overnight at 4 °C.

After washing with TWEEN-20 washing buffer, peroxidase-conjugated anti-human IgG was added and incubated for 2 h at 4 °C. After another washing step, OPD solution (Sigma-Aldrich) was added according to the manufacturer’s instructions for 30 min at room temperature (RT). Finally, the stop solution was added and optical density (OD) was measured within 30 min at 492 nm. Measurements were performed in triplets. Acetylcholine receptor antibodies in pre- and post-treatment samples were determined by standard radio immune precipitation assay.

### Immunohistochemistry using monomeric IgG

Dimeric IgG fractions were dialyzed against 10 mM acetic acid, pH 4.0, at 4 °C for 24 h and stored at −20 °C until further use.

Ten-micrometer sections from frozen rat sciatic nerve samples were cut on a cryostat and collected on Superfrost® Plus glass slides (Thermo Scientific). Following incubation at −80 °C overnight, sections were thawed at RT for 1 h in a dry chamber. After 1-h incubation in blocking solution (PBS containing 10 % normal goat serum and 0.4 % Triton X-100), sections were incubated overnight in a humidified chamber at RT with antibodies and developed with appropriate secondary antibodies. The following antibodies were used: monoclonal mouse anti-myelin basic protein (Abcam, 1:500) and monoclonal mouse anti-beta-III tubulin (Abcam, 1:500). For immunofluorescence, we used fluorescein antibody (horse anti-mouse IgG, 1:200; goat anti-human IgG). Sections were co-stained with Hoechst 33342 (Thermo Scientific) and mounted with Fluoromount-G™ (SouthernBiotech).

### Statistical analysis

Differences between serum samples and cell samples before and after treatment were analyzed by *t* test (two groups), and data were checked for normality using the Kolmogorov-Smirnov test. A *p* value <0.05 was considered statistically significant. Data is presented as mean +/- standard deviation.

## Results

### Analysis of IgG dimer content before and after treatment with IVIg

**Fig. 1:**
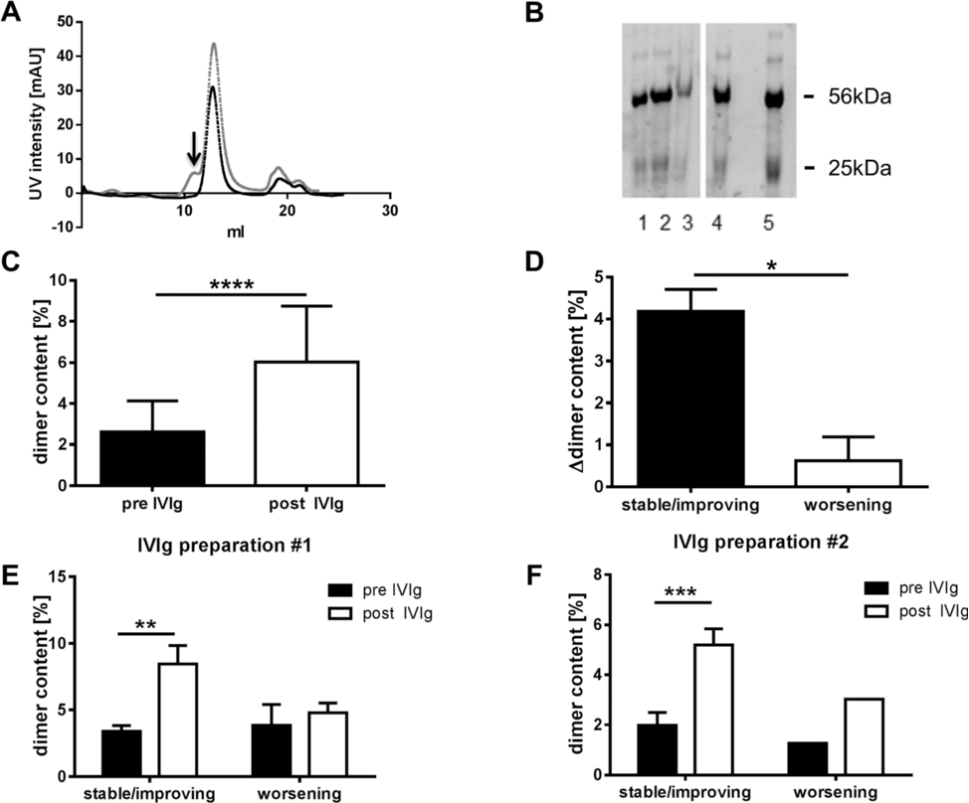
**a** Chromatography elution profile of representative pre-IVIg (*black*) and post-IVIg (*grey*) treatment sera. Dimeric IgG fractions (*arrow*) can be detected in post-IVIg treatment samples. **b** Gel electrophoresis of purified IgG fractions. Heavy chain (*56 kDa*) and light chain (*25 kDa*) of CIDP patient monomeric IgG fraction pre-IVIg (*1*) and post-IVIg (*2*) treatment could be detected. IgG purity in CIDP patient dimeric IgG fraction post-IVIg is also shown (*3*) compared to IgG of healthy control (*4*) and commercial IVIg preparation (*5*). **c** Dimer content of CIDP patients (*n* = 16) pre- and post-IVIg treatment. Dimer content is significantly increased after IVIg treatment. Change of dimeric IgG fraction in CIDP patients (*n* = 16) pre- and post-IVIg treatment. **d** CIDP patients with stable/improving course of disease show significantly higher dimeric IgG compared to worsening patients. **e**, **f** CIDP patients with stable/improving clinical course of disease after receiving either IVIg preparation #1 (**e**) or #2 (**f**) show a significant increase of dimeric IgG fraction post-treatment (*p < 0.05; **p < 0.01; ***p < 0.001); ****p < 0.0001)

We first analyzed the dimer content in serum IgG fractions before (pre) and after (post) IVIg infusion in patients with CIDP, MG, and MFS. Dimeric IgG fractions could be detected in most serum samples and varied between 0 and 11.3 % of total IgG (Fig. 1a, b). In CIDP patients, there was no association between IgG dimer levels pre- and post-IVIg treatment and age, body weight, or disease severity. IgG dimer levels were significantly higher in post- compared to pre-infusion samples (dimer content pre-IVIg 2.6 % ± 1.5, post-IVIg 5.8 % ± 2.9, Fig. 1c). We also measured the percentage of IgG dimers in five controls and found similar IgG dimer levels compared to CIDP patients (dimer content 2.4 ± 1.3 %). Further, we assessed the dimer content in different lots of two commercial IVIg preparations (Privigen®, CSL Behring and Gamunex®, Grifols) that were used in our patients and found higher values (4.0 % ± 2.3 and 4.9 % ± 1.5, respectively) compared to the average percentage in healthy controls and pre-treatment serum samples from CIDP patients.

### High IgG dimer levels after IVIg treatment are associated with disease stabilization

To assess the utility of IgG dimer levels as a surrogate marker for treatment response to IVIg, we grouped our cohort of CIDP patients according to their clinical response to IVIg treatment (stable/improving vs. worsening) and calculated the change of IgG dimer values in those two groups. CIDP patients who showed improvement or stabilization of the disease course during IVIg treatment (*n* = 13) had significantly elevated post-IgG dimer levels compared to those who worsened under IVIg treatment (*n* = 3, Fig. 1d). This difference could be detected irrespective which of the two available IVIg preparations were used (Fig. 1e, f).

### Autoantibody testing in newly formed dimeric IgG after IVIg treatment

There is strong experimental evidence that IgG dimers, which are detectable in pooled human IgG fractions from different individuals, consist of Id-anti-Id antibody pairs. Therefore, it is likely that in CIDP, post-treatment IgG dimers may also contain Id-autoantibodies and their anti-idiotypes. This hypothesis cannot be directly validated in CIDP, since the target antigens of the presumed autoantibodies in CIDP are unknown.

**Fig. 2:**
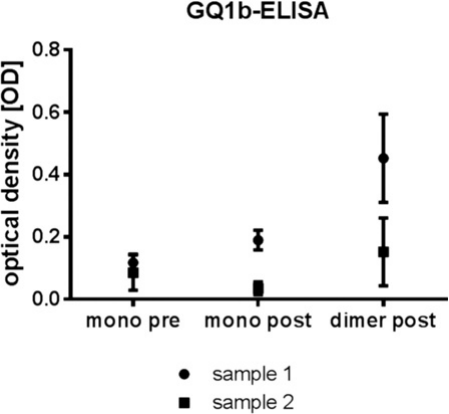
Anti-GQ1b antibody ELISA of samples from MFS patients. Anti-GQ1b-antibodies are detectable in monomeric IgG fractions pre- and post-IVIg treatment and in dimeric IgG fraction post-IVIg treatment

To further characterize the nature of IgG dimers that emerge after IVIg infusion, we collected monomeric and dimeric IgG peaks from two patients with MFS and from one patient with MG who were treated with IVIg. Patients #1 and #2 were seropositive for anti-GQ1b IgG antibodies, and patient #3 had serum IgG antibodies against acetylcholine receptors (71 nmol/l). We anticipated that IVIg dimers, which occur after IVIg treatment as a result of Id-anti-Id binding, should contain measurable amounts of those autoantibodies. Therefore, we re-monomerized the dimer fraction by dialyzing against 10 mM acetic acid at pH 4.0 and tested pre-IVIg treatment monomeric IgG and post-IVIg treatment monomeric and dimeric IgG fractions for immune reactivity against GQ1b and AchR, respectively. Monomeric and dimeric IgG fractions of the patient with myasthenia displayed reactivity against AchR (pre-monomeric 969 ± 10.1 cpm, post-dimeric 127 ± 6.5 cpm, post-monomeric 547.2 ± 16 cpm). Reactivity against GQ1b could be shown in monomeric IgG fractions pre- and post-IVIg treatment as well as to a higher extent in dimeric IgG fraction post-IVIg treatment (Fig. 2), suggesting that IgG dimers which occur after IVIg treatment in those two patients are formed by complexes that include autoantibodies and their presumed anti-idiotypes in IVIg preparations.

### Post-IVIg dimeric IgG fractions show immune reactivity against peripheral nerve fibers

**Fig. 3:**
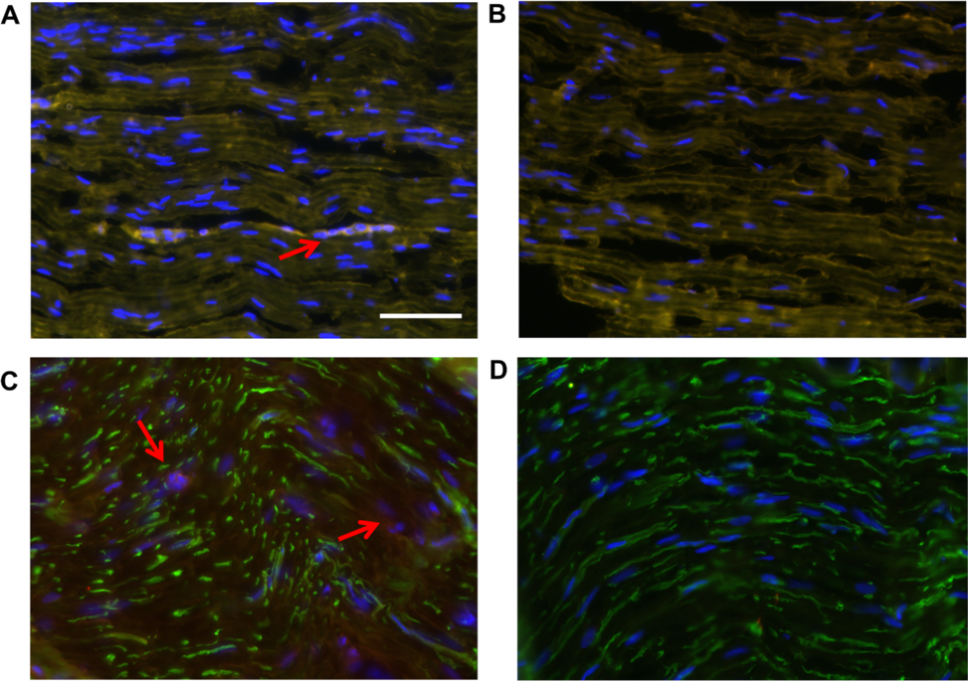
Monomerized dimeric IgG fractions of CIDP patients stained axonal and myelin structures of the peripheral nerve. **a** Double immunofluorescence labeling with Hoechst counterstain (cell nuclei, *blue*) revealed binding of monomerized dimeric IgG fractions (*red*) from CIDP patients to myelin basic protein (*green*). **b** Control IgG showed no specific binding. Also, double immunofluorescence labeling with Hoechst counterstain (cell nuclei, *blue*) showed binding of monomerized dimeric IgG from CIDP patients (*red*) to axonal nerve structures (beta-III tubulin, *green*) (**c**), whereas no binding was observed in control staining (**d**) (*scale bar* 50 μm)

Based on our experiments using GQ1b and AchR antibody-positive sera, we further anticipated that IgG dimers that occur after IVIg treatment in CIDP patients may also contain autoantibodies and their IVIg-derived anti-idiotypes. We therefore used post-IVIg treatment-derived IgG dimers for screening for autoimmune reactivity against neuronal or Schwann cell epitopes in peripheral nerve fibers. Four out of ten tested dimeric IgG samples stained axons and/or myelin of peripheral nerve fibers (Fig. 3).

This immunofluorescence was not detected in monomeric or dimeric IVIg fractions, pre-dimeric IgG, or from post-dimeric IgG derived from patients with MFS or MG. Pre-treatment of tissue samples with methanol/chloroform abrogated staining with dimeric IgG, indicating that those epitopes are membrane associated. From these data, we conclude that dimeric IgG contains antibodies that may recognize epitopes expressed on peripheral axons and Schwann cells.

## Discussion

Our study is the first that characterizes the nature of dimeric IgG fractions that emerge during treatment with IVIg in an immune-mediated neurological condition. We demonstrate here that IVIg treatment induces the formation of variable amounts of IgG dimers in CIDP patients. The amount of newly formed IgG dimers in vivo is associated with clinical stabilization and/or improvement during IVIg treatment.

Consistent with previous studies that reported only low levels of naturally occurring IgG dimers in healthy individuals [21, 24, 25], we found that the overall amount of IgG dimers in serum from controls and CIDP patients was considerably small. In contrast, commercial IVIg preparations contained higher amounts of IgG dimers. It is well known that pooled IgG from different donors contains usually higher amounts of IgG dimers; the amount of IgG dimers in commercial IVIg preparations varies between 5 and 15 %, depending on age, storage, formulation, and presence of chemical stabilizers [18, 19, 26].

The intravenous application of polyclonal IgG (IVIg) resulted in a marked and rapid increase of IgG dimer serum levels after treatment in most of our patients. Those levels differed and were mostly higher as one would expect by simply adding the percentage of IgG dimers that are present in commercial IVIg preparations to the pre-existing IgG dimer amount. Therefore, it must be concluded that IVIg infusion results in the spontaneous formation of novel immune complexes in vivo.

The association of high IgG dimer levels with clinical stabilization in CIDP can be explained by interaction and subsequent neutralization of autoantibodies with anti-idiotypes that are present in IVIg preparations. IVIg contains anti-Id against a broad range of autoantibodies including anti-phospholipid [14], anti-DNA [15], anti-thyreoglobulin [27], and anti-neutrophil cytoplasmic antigen antibodies [28]. Those anti-Id interact with variable regions of other antibody molecules to form Id-anti-Id complexes, which are detectable in dimeric IgG fractions [16, 21]. A direct proof that IgG dimers derived from CIDP patients contain Id-anti-Id complexes would be desirable but cannot be provided due to the lack of commonly occurring, disease-specific autoantibodies in CIDP [29, 30]. However, our studies with post-treatment samples from two patients with MFS and one patient with MG support this concept and provide evidence that IVIg contains also anti-Id against anti-GQ1b and anti-AchR antibodies, respectively. It further argues against the possibility that the IgG dimers in post-IVIg treatment samples contain exclusively IgG aggregates from IVIg preparations.

Although some of the monomerized IgG showed immunoreactivity against epitopes expressed on Schwann cells and peripheral neurons, the overall number of available samples was yet too small to define phenotype-specific staining patterns. This is supported from recent serological studies that reported novel disease-specific antibodies only in very small proportions (8.6 and 4.2 %) of cohorts of 46 [31] and 119 [32] CIDP patients. However, our immunolabeling studies with re-monomerized IgG fractions provide proof of concept that Id-anti-Id complexes that occur after IVIg treatment could be used as a novel approach to screen samples from CIDP patients in order to identify disease- or subgroup-specific autoantibodies. This approach is quite different from previous, mostly unsuccessful antibody screens that used whole serum fractions from CIDP patients [29, 30, 33]. The fact that a considerable proportion of patients’ IgG showed reactivity against axonal tissue or Schwann cells may indicate an increased sensitivity of the strategy to use dimer IgG fractions for antibody testing. However, further validation studies with a larger cohort of patients are necessary to independently confirm the usefulness of IgG dimer quantification as a putative biomarker for IVIg treatment in CIDP.

Limitations of our study are a considerably small number of patients, particularly the proportion of CIDP patients who worsened during IVIg treatment. In addition, the patients in our cohort were only followed over a time period of 6 months which restricts conclusions about the utility of IgG dimers to serve as a putative biomarker for CIDP. Future studies are also necessary to determine dimer formation in IVIg-treatable conditions that are not associated with autoantibodies, such as primary immunodeficiency.

Nevertheless, the results of our study support the notion that anti-Id antibodies contribute to the beneficial effect of IVIg treatment in CIDP. Furthermore, the quantification of IgG dimer levels could be a feasible approach to identify IVIg-responsive CIDP patients. Further assessment of pre- and post-treatment IgG dimer levels in larger patient cohorts is warranted to explore their utility so serve as a potential biomarker for treatment response to IVIg in CIDP patients.
